# Amur Softshell Turtle (*Pelodiscus maackii*) Population Size, Structure, and Spatial Distribution

**DOI:** 10.3390/ani15020255

**Published:** 2025-01-17

**Authors:** Xiaochen Hou, Haitao Shi

**Affiliations:** 1 School of Life Science, Tonghua Normal University, Tonghua 134001, China; 20211071300001@hainnu.edu.cn; 2 Ministry of Education Key Laboratory for Ecology of Tropical Islands, College of Life Sciences, Hainan Normal University, Haikou 571158, China

**Keywords:** continuous-time capture–recapture method, closed population, population size, conservation, amur softshell turtle

## Abstract

Freshwater turtle species preservation relies on understanding their population dynamics and geographical distribution. Amur softshell turtles (ASTs [*Pelodiscus maackii*]) are poorly protected due to insufficient awareness and the population in Northeastern China has experienced a steep decline compared to previous years. This study aims to investigate the population density and structure of ASTs in the Jewellery Island area of the Ussuri River in Northeast China using continuous-time capture–recapture methods in closed populations. A three-month mark–recapture study was conducted in 2022, resulting in 35 juvenile captures, including 12 recaptures from 23 marked individuals. The estimated population size in the study area was 40.79 ± 9.75 (95% confidence interval, 95% CI = 27–65), translating to 0.663 ± 0.158 turtles/ha (95% CI = 0.44–1.06 individuals/ha). Approximately 35.4–85.2% of the estimated population was marked. Additionally, we explored the influence of environmental variables on turtle distribution by dividing the surveyed sites into seven sections based on their natural characteristics. The ASTs distribution inferred from trapping successes was considerably different among sections, with most turtles (91%) captured at the vegetated shoreline and in water channel 2. This research offers essential baseline data to support future assessments of ASTs population on a larger scale and to inform the development of conservation strategies.

## Simple Summary

In this study, we investigated the population size of Amur softshell turtles (*Pelodiscus maackii)* around Jewellery Island, a river island in the mid-stream of the Ussuri River using continuous-time capture–recapture methods, and analyzed the spatial distribution of Amur softshell turtles based on cage trapping results. The results revealed the disturbingly low population density of Amur softshell turtle in this area, highlighting the urgent need for targeted conservation efforts. Our spatial distribution study indicates that juvenile turtles predominantly inhabit still-water channels and vegetated shoreline areas, emphasizing the critical importance of these habitats. This study establishes a foundational framework for future population assessments and pinpoints vital conservation areas. Our results fill a gap in the knowledge of the natural history of this endangered species.

## 1. Introduction

The Amur softshell turtle (*Pelodiscus maackii*, [ASTs]) is a freshwater turtle species that was once distributed throughout Northeastern China, the Russian Far East, the Korean Peninsula, and Japan [1,2]. The species is believed to be the largest member of the genus *Pelodiscus*. However, this turtle species remains poorly studied due to the sharp decline in recent decades in Northeastern China owing to increased anthropogenic activities. The remaining ASTs population within the Chinese border was restricted to remote and unexplored areas. While there may be relatively robust populations in the Russian Far East, however, research on these populations remains limited and outdated, with a primary focus on population surveys. Although ASTs were described in the 19th century, information regarding their ecology, including current population status and spatial distribution characteristics throughout their habitat, is exceedingly scarce. The International Union for Conservation of Nature’s (IUCN) Red List categorizes ASTs as “Data Deficient”, making it challenging to assess their conservation status accurately.

Estimating population density is necessary for effective management and conservation initiatives [3,4]. Unfortunately, research on the ecology of ASTs is relatively scarce. Some work has been conducted by scientists in Russia and the former Soviet Union in Russia’s Far East area, but many of these studies are difficult to access due to their age and being published in Russian. Most of these articles focus on surveys and population descriptions of ASTs distribution within Russia [5,6,7,8]. Notable ecological studies include Adnagulov et al. [9], who examined ASTs population in the Bidzhan River, a tributary of the middle Amur River on the Russian side. They identified seven habitats and concluded that three of them—deep sections of riverbeds and arms, gulfs and floodplain lakes, and nesting spits—are critical for the survival of the local ASTs population. Similarly, Maslova [10] studied ASTs population in the middle Ussuri River and its tributaries (Bol’shaya Ussurka and Malinovka Rivers) on the Russian side. This study highlighted the population as the most endangered and heavily impacted by poaching from fishermen on the Chinese side. Coincidentally, our study area is located on the Chinese side of this region. Although some research has been conducted, the overall number remains quite limited, particularly concerning the ecological aspects, and there is currently no detailed investigation into ASTs population size estimation. In China, where ASTs face an imminent threat of extinction, their ecological characteristics remain poorly understood. The ongoing indiscriminate hunting, fueled by limited awareness and inadequate conservation efforts, underscores the urgent need for ecological studies, particularly those focused on assessing population status.

Being the sole turtle species native to Northeastern China and the Russian Far East [7,8], the ASTs is the top predator in local rivers and likely plays a crucial ecological role [11]. Sustaining a robust population may positively impact the health of local river ecosystems. Turtles require specific environmental conditions for their survival [12] and understanding their distribution and habitat preferences is crucial for identifying priority conservation areas. Thus, it is vital to investigate the environmental factors shaping the spatial ecology of turtles within their habitats [13]. Environmental conditions also strongly influence other turtle population parameters, such as population density, birth rate, mortality, and sex ratio [14]. The correlation between population density and local environmental conditions helps define how space is utilized and what constitutes “suitable” habitats.

Accurate data on the abundance and distribution of this endangered species is critical for designing effective conservation programs in the region. This study aimed to assess the abundance and density of ASTs in a region south of Jewellery Island in the Ussuri River, examine distribution patterns and the environmental factors influencing their active range, in order to establish a reference framework for a broader scale population assessments, and provide essential baseline data to support the creation of future conservation management strategies.

## 2. Materials and Methods

### 2.1. Study Area

**Figure 1:**
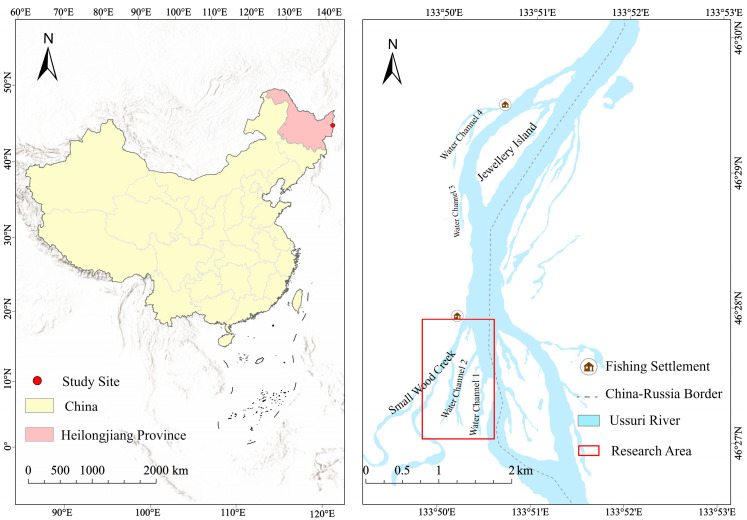
(**Left**) Map of Heilongjiang Province, China. The study site is marked by a red dot. (**Right**) The study area and its surroundings on the Ussuri River. A red square indicates the sampling area. The map was constructed using ArcGIS Pro 3.2 [15].

**Figure 2:**
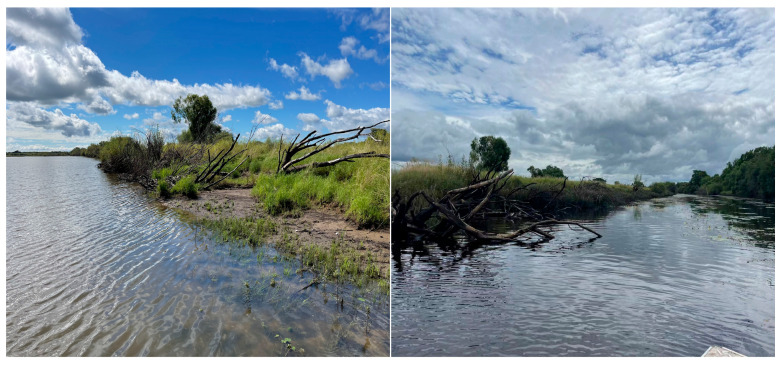
*Pelodiscus maackii* habitat at the Ussuri River, China. (**Left**) Undisturbed banks of the main river course. (**Right**) View of water channel 2. Photographs by Xiaochen Hou.

The study was conducted near the Jewellery Island (45°52′–45°17′ N, 133°28′–133°47′ E) in the Ussuri River, Heilong Jiang Province, China (Figure 1). Sampling was conducted in the region south of Jewellery Island, encompassing two primary water channels and a tributary known as Small Wood Creek. Shallow water areas (<1.5 m) were near the shoreline and composed most of water channel 2, while the water depth in water channel 1 and Small Wood Creek was relatively deep (<2.5 m and <3 m, respectively). Most of the main watercourse of the Ussuri River ranged between 3 and 6 m deep. The bottom was mainly composed of mud and sand, with a prevalence of sandy sediment at the shoreline of the main watercourse and sandy gravel in the upper sections of the Small Wood Creek. Predominant vegetation types along the shoreline included willows (*Salix* sp.), reeds (*Phragmites* sp.), and sedges (*Cyperus* sp.) (Figure 2). Most of the study area showed few signs of human activities, with only a small altered portion of the shoreline characterized by piles of large boulders in the south. The area is bordered by extensive wilderness with minimal impact from human activity, although fishermen frequently visit the area. A settlement of fishermen is located in the northern part of the sampling area near the mouth of the Small Wood Creek. The Ussuri River serves as the border between Russia and China. Owing to jurisdictional limitations, our study was restricted to the Chinese side of the river, and our findings mainly represent the situation on this side of the Ussuri River.

### 2.2. Field Survey

**Figure 3:**
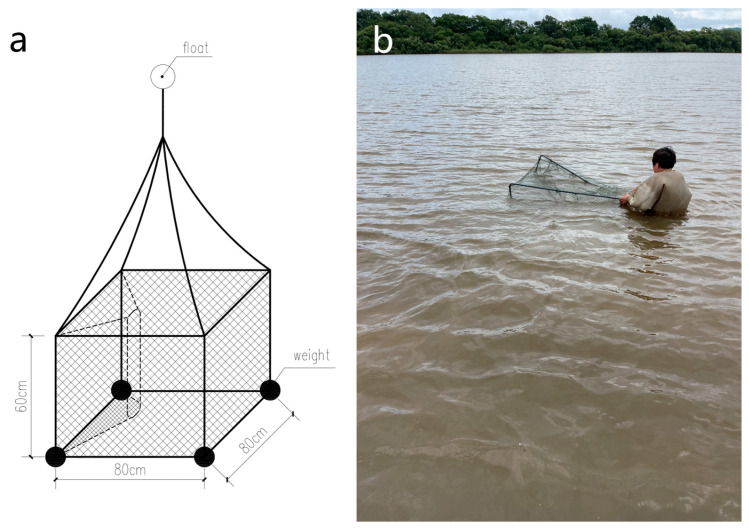
(**a**) Schematic drawing of cage trap design. (**b**) Cage deployment.

A capture-mark-recapture study was carried out during the summer months of 2022 (from late June to early October), coinciding with most peak activity periods of ASTs in this region. In the sampling area, 31 specially designed traps were strategically distributed according to distribution of habitat types in the Ussuri river and its immediate tributaries. Traps were 80 × 80 × 60 cm, with an 80 × 60 cm entrance constructed to accommodate the largest adult turtle (Figure 3a). Each time a trap was deployed, a Garmin 60 CSX global positioning system (GPS map 78 s, Garmin International Inc., Olathe, KS, USA) was used to record its positions.

We measured several habitat features at each trapping location that could potentially influence trapping success. These include water depth (m), water velocity (m/s [measured at 20 cm water depth]) (LS300-A, Fuzhou Lestar Information Technology Co., Ltd. Fuzhou, China), water plant coverage (% [percentage coverage of water plant in a 2 m × 2 m square riverbed centered around the trapping location]), woody debris (% [approximate volume percentage of submerged wood in a 2 m water column. centered around trapping location and measured on the day of trap deployment]), canopy (% [estimated from the shadows cast on the water surface and the proportion of the surface covered by overhanging vegetation in a 2 m × 2 m frame]), and sand content of the substrate (% [sand percentage in a 200 g samples of substrate at each trapping location]). These environmental variables were assessed in the field, with the exception of sand content of the substrate, which were evaluated upon returning to base.

Carp meat and guts were used as bait for turtles. Traps were checked daily to reduce the risk of trapped turtles drowning. Upon capturing a new specimen, the trapping location, trapping time, body mass, and sex were recorded. The body mass of each turtle was measured to the nearest 1 g using an electronic scale (Meldrum Scale Company, Sandy, UT, USA). We utilized multiple characteristics, similar to those used for other softshell turtle species (e.g., tail length), to distinguish between sexes. The existing literature lacks specific guidelines for determining the size at sexual maturity of ASTs. However, through observation of fishermen processing captured ASTs in nearby towns, we noted that female ASTs weighing approximately 900 g tend to have developed ovaries, whereas males weighing approximately 800 g have fully developed secondary sexual organs. Consequently, this empirical criterion was adopted as the method for assessing the sexual maturity of ASTs in the absence of established standards.

For identification, a unique mark on the outer carapace edge was created using a hole punch [16]. In the event of a recapture, we referred to the records to identify the turtle and record the trapping location and time. After recording, the turtles were released near their capture locations. We defined the initial capture as the first time a novel animal was caught. A recapture was defined as the capture of a previously marked turtle. The observations for each turtle were ranked in chronological order. Throughout the study period, the marking wounds have fully healed, showed no signs of infection and all the turtles remained in apparent good health with no fatalities. All turtles were released at the conclusion of the study. This study was approved by the Fisheries Department of Hulin County, Hulin, China.

**Figure 4:**
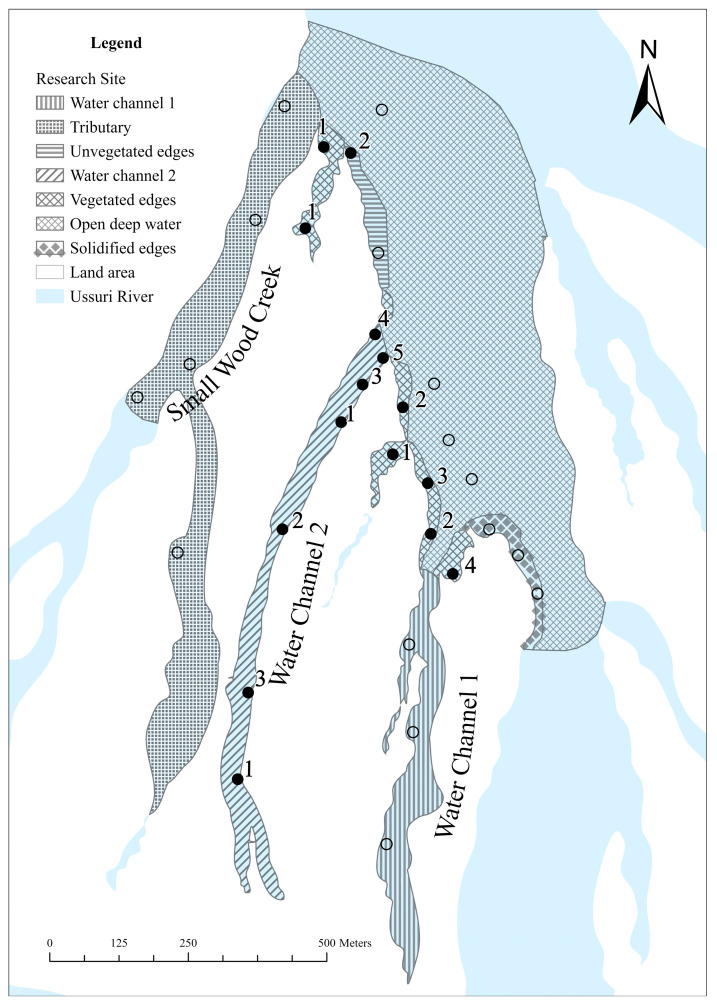
Map of the study site highlighting the seven major habitat sections identified during the study. Solid dots represent locations where capture successes occurred, with numbers denoting the total number of successful captures (including recaptures). Hollow dots mark locations where no captures were recorded. The map was constructed using ArcGIS Pro 3.2 [15].

We constructed a habitat map based on a field survey that divided the study area into seven major habitat sections, each encompassing trapping locations to analyze the spatial distribution of ASTs in this region (Figure 4). Across sections of varying size, 6–10 random 1 m × 1 m sample plots were randomly selected. We chose six variables, including water temperature (°C [average water temperature was measured at noon over a fixed 10-day period at each sample plot at 50 cm depth]) (testo 635-2, Testo SE and Co. KGaA, Titisee-Neustadt, Germany), water velocity (m/s [measured at 20 cm water depth]) (LS300-A, Fuzhou Lestar Information Technology Co., Ltd. Fuzhou, China) water plant coverage (% [percentage coverage of water plant in the riverbed of each sample plot]), woody debris (% [approximate volume percentage of submerged wood in a 1 m water column at each sample plot]), canopy (% [estimated from the shadows cast on the water surface and the proportion of the surface covered by overhanging vegetation in a 1 m × 1 m frame]), and sand content of the substrate (% [sand percentage in a 200 g sample of the substrate at each sample plot]), selected based on preliminary assessments of differences between the sections.

### 2.3. Data Analysis

#### 2.3.1. Population Size Determination

A continuous-time capture–recapture model was used to estimate the population size (*N*). As we accurately identified each specimen and observation occasion, the collection $t=(t_{1}^{T},\cdots ,t_{N}^{T}{)}^{T}$ can be confirmed. *T* means the total number of observations throughout the study period. The vector ${t_{i}=(t}_{i1},\cdots ,t_{ici})$ gives the ordered capture times for each individual i, $t_{ij}$ denotes the jth observed time of capture for the ith individual: $\left(i=1,\cdots ,N,j=1,\cdots ,c_{i}\right)$. Two important summaries: $c=(c_{1},\cdots ,c_{n})$*^T^*, the number of observations for individual i in (0, τ), denoted as $c_{i}$, τ means total trapping duration; $y=(y_{1},\cdots ,y_{h})$*^T^*: the identity of the hth observation, denoted as $y_{h}$ [17], can be deduced from t.

Various models can be fitted, given the availability of c and y. We considered and ran the tests for the heterogeneity *M**th*** model, where the total catchability varied by individual, and the behavioral *M**_tb_*** model, where previous captures influence current intensity [17]. All the models were fitted using the algorithms described by Schofield et al. [17]. We estimated the posterior distribution for the model *M**_t_*** (assuming no heterogeneity or behavioral effects) using *M* = 120. M is an assumed upper population size value. For the *M**th*** and *M**_tb_*** models, we opted for consistent prior specifications, setting ***θ*** = 4.6, 9.2, and 50 for the *M**th*** model and ***θ*** = 4.2, 11.4, and 48.3 for the *M**_tb_*** model, which is consistent with the examples outlined by Schofield et al. [17]. The greater the ***θ*** value, the less pronounced the heterogeneity and behavioral effects, indicating a model resembling the *M**_t_*** model. Three Markov chains were sampled, each with 26,000 samples; the first 1000 were discarded as burn-in after optimizing the algorithm. The models were constructed in R version 4.2.0 [18], utilizing the R package ‘ctime’ [17]. The population density was calculated by dividing the population size (N) by the area (ha). The study area was calculated using ArcGIS Pro 3.2 (Esri, Redlands, CA, USA) [15]; incorporating the cage locations, and covering the entirety of water channels 1 and 2, along with a section of Small Wood Creek. The boundary of open deep water was established by an extension of 140 m from the outermost caging point in open water (Figure 4). This determination was based on the average of the maximum daily distances traveled by juvenile ASTs, as revealed in Hou [19].

#### 2.3.2. Spatial Distribution Pattern Analysis

Differences in environmental characteristics were assessed based on variables measured at each 1 m × 1 m sample plot between sections using Non-metric Multidimensional Scaling (NMDS). This analysis was performed using the ‘metaMDS’ function within the R package ‘vegan’ [20]. We utilized χ^2^ goodness-of-fit test to understand potential distribution patterns among the sections based on trapping successes [21,22,23]. The confidence intervals were determined using Bonferroni z-tests [21,23]. We constructed a generalized linear model using Poisson distribution and log link function to investigate the potential correlation between environmental variables and the total number of trapping successes at each trapping location. Zero inflation was assessed in our data using the R package ‘performance’ [24]. Multicollinearity was evaluated using a variance inflation factor (VIF) applied to the explanatory variables of the model using the ‘vif’ function from the R package ‘usdm’ [25]. A VIF > 10 indicates problematic multicollinearity [26], necessitating a reassessment of the variables. Considering the number of habitat variables measured and our relatively small data size (31 trapping locations), a variable selection procedure was implemented using the ‘all possible subsets regression’ function in the R package ‘olsrr’ [27] to reduce variable numbers to avoid overfitting [28]. We examined dispersion (residual deviance/degrees of freedom) using selected habitat variables in Poisson regression. The result revealed that our data were under dispersed. Therefore, we employed Quasi-Poisson regression [29] to reduce the standard error. The analysis was performed using the ‘glm.nb()’ function within the R package ‘MASS’ [30]. Subsequently, we verified the residuals to ensure the model assumptions were satisfied. Finally, a hierarchical partitioning analysis was implemented using the R package ‘hier.part’ [31] to estimate the contribution of each variable. All analyses were performed using R version 4.2.0 [18].

## 3. Results

### 3.1. Population Size Estimation

Sampling was performed between 3 July and 1 October 2022 (>91 days [2821 trap-days]), during which 35 individuals were captured and 12 recaptured. Of the 23 marked turtles, 4 were male (17.4%), 1 was female (4.3%), and 18 (78.3%) were juveniles of indeterminate sex. Every trapped individual and trapping event was documented (Table 1). The adopted marking system was effective, with no instances of misidentifications or loss of marks recorded throughout the study period. The surface area of the sampling area was approximately 61.54 ha.

**Figure 5:**
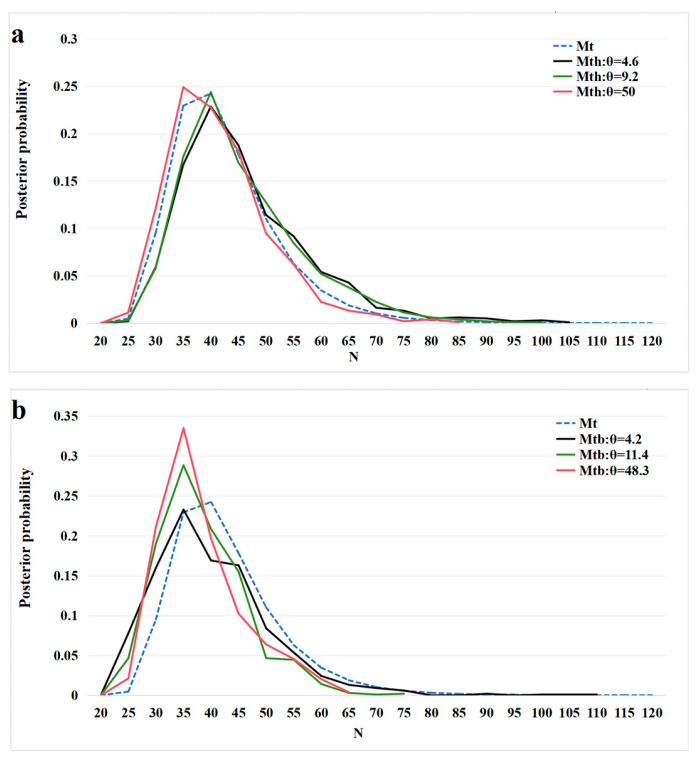
(**a**) Posterior densities of N for the data using the *M**_t_*** and *M**th*** model. (**b**) Posterior population size of *N* for the data using the *M**_t_*** and *M**_tb_*** model. ***θ*** denoted the proposed default prior choice [17].

As c and y can be inferred from Table 1, we were able to fit all models. Tests for heterogeneity and behavioral effects revealed no evidence of either in our data. The heterogeneity test had a *p*-value of 0.3074, “where a *p*-value < 0.05 denoted a higher variability in the observed counts than that expected in its replications under the *M**_t_*** model”. The behavioral test had *p*-values of 0.6461, 0.5214, and 0.6249 for K = 1, 3, and 5, respectively, “where a *p*-value < 0.05 would indicate that the same individual was captured more often than expected in the next K observations if the *M**_t_*** model was correct” [17]. The posterior distribution of N demonstrated a relatively consistent pattern across different models with different prior specified ***θ*** values (Figure 5). There was a significant overlap, and the modes under the heterogeneity model were within the range of values under the *M**_t_*** model (Figure 5a). The models differed in the length of the right tail. The relative similarity of the posterior densities supported the heterogeneity test results. The posterior for the *M**_tb_*** model (Figure 5b) was slightly shifted to the left compared with that of the *M**_t_*** model, indicating increased prior sensitivity in the *M**_tb_*** model. Despite less significant overlap between the models and greater variations in the upper limits of the right tail, there was a considerable overlap, indicating that the results were consistent with the previous test. Based on the above analysis, the outcomes of the *M**_t_*** model seem plausible. The estimated size of the ASTs population in this region was 40 ± 9.75 (95% confidence interval (CI) = 27–65 [Figure 5]). Therefore, the population density in the region was 0.663 individuals/ha (95% CI = 0.44–1.06 individuals/ha).

### 3.2. Spatial Distribution Pattern

**Figure 6:**
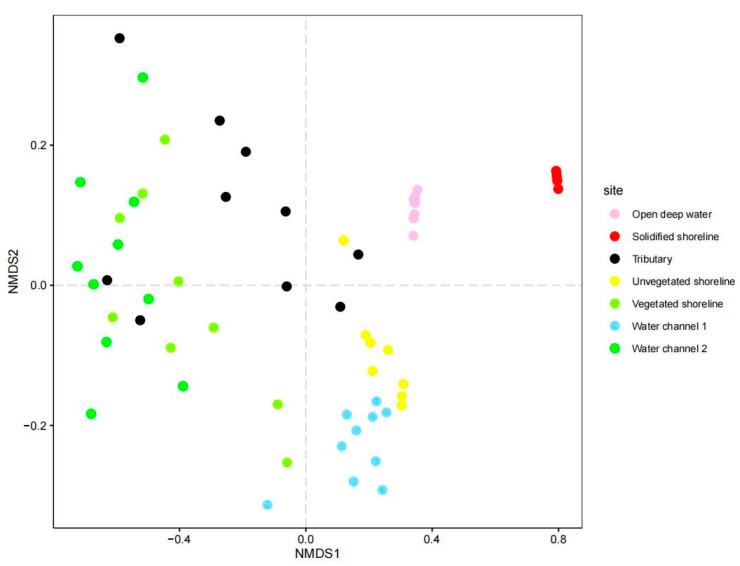
Differences in environmental variables (water temperature [°C], current [m/s], water plant coverage [%], woody debris [%], canopy [%], and sand content of the substrate [%] across the seven sections (Figure 4) visualized using the Non-metric Multidimensional Scaling (NMDS).

Differences in environmental characteristics among the sections were observed. Sample plots from water channel 2 and the vegetated shoreline were nested together with a few sample plots in the tributary. Sample plots of the other sections were generally separated (Figure 6). Water temperature was consistent across all sections except in the tributary, which was noticeably cooler. The current (m/s) and sand content of the substrate (%) varied considerably between sections, with open deep water having the strongest current (m/s) and the unvegetated shoreline having the highest sand content (%). Water plant coverage (%), canopy (%), and woody debris (%) were relatively high in the tributary, vegetated shoreline, and water channel 2; however, the values ranged widely. These three variables were generally low in the other sections (Figure 4). The uneven trapping successes indicated that the distribution of ASTs was not proportional to section availability ($\chi_{1}^{2}$ = 19.63, *p* < 0.001). The Bonferroni confidence intervals (95%) indicated that the proportion of water channel 2 used by ASTs exceeded the expected proportion. Although most (90%) of the Bonferroni confidence intervals (95%) were above the expected proportion of trapping successes for vegetated shorelines, this was not statistically significant. Analysis of the ASTs distribution in other sections was not feasible owing to insufficient trapping successes (<5); however, a zero-trapping success unequivocally indicated negative use (Table 2). Three selected habitat variables, water plant coverage (%), in water (%), and canopy (%) were retained from the variable selection procedure. A Quasi-Poisson regression revealed significant correlations between woody debris (%) and canopy (%) with trapping successes (Incidence Rate Ratio [IRR] = 1.06, *p* < 0.001, 95% CI = 1.03–1.10 and IRR = 1.03, *p* = 0.003, 95% CI = 1.01–1.05, respectively; Table 3). Hierarchical partitioning analysis demonstrated that woody debris (%) was the most important variable explaining total trapping success at each location, contributing 53.40% for the percentage likelihood (I% [Table 4]). ASTs were primarily captured in shallow waters with an abundance of submerged wood.

## 4. Discussion

### 4.1. Population Size and Structure

Various methods have been utilized for estimating animal population density, with the most common being the discrete sampling capture–mark–recapture model, such as the widely used Jolly–Seber model [32,33]. While extensively documented [34,35,36], these methods require discrete sampling occasions. This requirement may be satisfied by separate trapping sessions [37,38,39]. While feasible with ample research time, as in sampling periods spanning consecutive years [38], it poses challenges for short-term sampling, such as within a single active season, as this may be too brief to discretize effectively (as observed in this study). Contrastingly, the recently developed continuous-time models [17,40,41,42], which involve sampling animal populations in near-continuous time, provide new approaches for population evaluation despite their increased complexity.

To mitigate potential bias introduced by different capturing techniques [43], we constructed traps capable of capturing turtles of all sizes, aiming to prevent skewed captures that might target specific size groups of turtles. Significantly, no adults weighing > 900 g were captured. This phenomenon may stem from two potential factors, either individually or in combination. First, the intensive harvesting of ASTs on the Chinese side. Given the tradition of consuming soft-shelled turtles in China and the high market value of large ASTs, which can sell for up to USD100 per kilogram, fishermen employ advanced and highly efficient gear to capture them. And this practice operates without any regulations or restrictions by now. Smaller individuals, on the other hand, may evade capture by seeking refuge in water plants or woody debris, or are discarded after being captured due to their low market value, allowing them to sustain a limited presence. Maslova [10] also highlighted in his study that the ASTs population in the midstream of Ussuri River has been significantly affected by fishing activities from the Chinese side.

We noted that the population surveys conducted by Russian and Soviet researchers, showed a healthy ASTs population typically found in tributaries deeper within Russian territory, where the influence from Chinese fishermen is minimal, as Russians generally neither catch nor consume ASTs. In contrast, our two-year surveys along the Chinese side of the Ussuri and Amur rivers revealed severe depletion of ASTs population due to the impact of the expansion of human industrial and agricultural activities and long-term, unregulated poaching. While small, scattered populations persist in the main channels of the two rivers, the tributaries on the Chinese side have largely been rendered devoid of ASTs, with many populations entirely extinct.

This severe population decline appears to be the most plausible explanation for the scarcity of large ASTs in the study area. Alternatively, it is also possible that this region does not provide a preferred habitat for adult ASTs. Based on this, we speculate that the capture of predominantly juvenile and small-sized ASTs during our surveys is not due to bias in our capture methods but reflects the absence of large ASTs in this region. Therefore, it is highly likely that our population estimates closely reflect the actual population size, rather than significantly underestimating it due to the absence of adult captures. This hypothesis is further supported by the catch records of experienced local fishermen. Despite this area being one of the few regions with a confirmed ASTs population in adjacent waters, large individuals are exceedingly rare and difficult to find. According to older fishermen; however, the area had a much richer population of ASTs 40 to 50 years ago, similar to other parts of the Ussuri River. This highlights the dramatic decline in ASTs population over the years, particularly on the Chinese side.

However, even if adults were captured, the assumption of a closed population was not satisfied (adults frequently use deep-water areas and migrate freely in and out of the study area according to Hou [19]). In such cases, alternative population estimation methods would be necessary. Our model, based on the closed population assumption, predicted juvenile ASTs population more accurately, as their movement is typically restricted to Stillwater channels and shallow nearshore waters Hou [19]. The open deep waters in the study area acted as a natural barrier, effectively isolating juvenile population, particularly over short periods. This behavioral trait of juvenile ASTs supports the closed population assumption.

The population density estimation of ASTs in this area falls within the lower range of values reported in published literature for other softshell turtle species (0.11–42/ha [Table 5]). The rationale for selecting our study area for population density examination was based on this body of water has the only confirmed ASTs population within a large stretch of the surrounding river. Therefore, we inferred that our study area is the preferred habitat for ASTs. Considering this, ASTs population would look even more precarious (China side).

### 4.2. Spatial Distribution Pattern

Almost all ASTs were captured on the vegetated shoreline and water channel 2, confirming the significance of these two areas for the survival of ASTs in this region. The Bonferroni confidence intervals (95%) indicated that the proportion of vegetated shorelines used was not statistically significant; however, this may be due to the excessive error introduced by the small sample size. NMDS revealed that the sample plots of the vegetated shoreline and water channel 2 had similar characteristics and were largely nested together, and both regions had relatively higher water plant coverage, woody debris, canopy, and low current. The woody debris was mainly comprising dead wood and some living tree branches and root systems. This environment may be a refuge and retreat for juvenile ASTs and hosts various small animals (including insect larvae, crustaceans, mollusks, snails, amphibians, and fish) [47,48], which are potential prey for the turtles [49]. Additionally, water channels and submerged wood-abundant vegetated shorelines provide some of the few slow-velocity water current bodies, apart from the main watercourse, that are essential for the well-being of softshell turtles [50].

We noted that the tributary had many similar suitable areas; however, no ASTs were caught here. We speculated that this might be largely because the Small Wood Creek tributary originated in mountainous areas, leading to cooler water temperature compared with other parts of the river. ASTs, as ectothermic animals, might be sensitive to temperature changes, prompting them to avoid cool-water zones for thermoregulation purposes.

Although water channel 1 provided slow-velocity water bodies, it had greater water depth, and the banks consisted of meadows without tree growth, resulting in little woody debris that would provide shelter and hiding places for juvenile ASTs. These factors were believed to be the main reasons for zero trapping success in this section. Quasi-Poisson regression and hierarchical partitioning analyses consistently underscored the significance of woody debris in predicting trapping success.

Overall, the substantial utilization of water channel 2 and vegetated shorelines strongly implies that these areas serve as superior habitats and resource-rich environments for juvenile ASTs. This insight emphasizes the importance of prioritizing the protection of similar regions in future conservation management efforts.

## 5. Conclusions

To the best of our knowledge, this study is the first to evaluate the population status of ASTs in the Ussuri River and to estimate the turtle population using the continuous-time capture–recapture method. Our study establishes foundational information and methodological frameworks applicable to research in various similar sections of the Ussuri River, providing a comprehensive approach to population studies that can facilitate future IUCN Red List evaluations for ASTs. Furthermore, our research reveals that ASTs population in the Ussuri River (China side) are both scarce and have undergone a significant decline compared to previous years. Additionally, their distribution is uneven and closely tied to specific habitats. Juvenile ASTs survive in undisturbed, shallow, and calm water bodies with healthy native plant communities and submerged wood. Consequently, safeguarding these critical areas from anthropogenic disruptions and reinforcing pertinent protective measures are imperative for ensuring the enduring survival of ASTs in the Ussuri River. Moreover, although the undisturbed and pristine stretches along the Russian riverbank may serve as a refuge for ASTs, the unregulated hunting of these turtles on the Chinese side of the river still poses a significant threat to the ASTs population in this region. Consequently, it is imperative to impose a comprehensive ban on the indiscriminate hunting and consumption of this species in China to prevent their further population decline.

## Figures and Tables

**Table 1:** Marked individuals and recapture occasions in the study area. Observation Occasion: ordered capture occasions for each individual turtle.

Turtle ID	Weight (g)	Sex	Observation Occasion *t = (t*_1_ … *t*_23_*)*
1	70	Juvenile	*t*_1_: 1, 6, 26
2	65	Juvenile	*t*_2_: 2, 10
3	358	Male	*t*_3_: 3
4	148	Juvenile	*t*_4_: 4
5	95	Juvenile	*t*_5_: 5,27
6	56	Juvenile	*t*_6_: 7, 16, 21, 28
7	120	Juvenile	*t*_7_: 8, 30
8	230	Juvenile	*t*_8_: 9
9	47	Juvenile	*t*_9_: 11
10	68	Juvenile	*t*_10_: 12
11	159	Juvenile	*t*_11_: 13
12	280	Male	*t*_12_: 14, 17, 29
13	38	Juvenile	*t*_13_: 15
14	52	Juvenile	*t*_14_: 18
15	46	Juvenile	*t*_15_: 19
16	680	Male	*t*_16_: 20, 33
17	69	Juvenile	*t*_17_: 22
18	155	Juvenile	*t*_18_: 23
19	450	Male	*t*_19_: 24
20	89	Juvenile	*t*_20_: 25
21	595	Female	*t*_21_: 31, 34
22	116	Juvenile	*t*_22_: 32
23	72	Juvenile	*t*_23_: 35

**Table 2:** Section selection of *Pelodiscus maackii*. (*pi*) ^a^ Bonferroni confidence interval (95%) of trapping success under the expected selection hypothesis. sp ^b^ significant selection for section type: + = significantly higher than expected; − = significantly below expectation; ns = not significant; * = unable to perform analysis owing to insufficient number of trapping successes (<5).

Section	True Proportion of Trapping Successes (*pi*)	Proportion of Trapping Locations (*pio*)	CI 95% (*pi*) ^a^	sp ^b^	True No. of Trapping Successes	Expected No. of Trapping Successes
Open deep water	0.00	0.130	0.00	−	0	4.5
Tributary	0.00	0.160	0.00	−	0	5.5
Water channel 1	0.00	0.100	0.00	−	0	3.4
Water channel 2	0.54	0.225	0.32 ≤ ***pi*** ≤0.77	+	19	8
Solidified edges	0.00	0.100	0.00	−	0	3.5
Vegetated edges	0.40	0.225	0.18 ≤ ***pi*** ≤ 0.62	ns	14	8
Unvegetated edges	0.06	0.060	*	*	2	2
Total					35	35

**Table 3:** Quasi-Poisson model for the correlation between the total trapping successes in each trapping location and environmental drivers (water plant coverage [%], woody debris [%], and canopy [%]). Acceptance level was α = 0.05.

Predictors of Catch Number			
Predictors	Incidence Rate Ratios	CI	*p*
(Intercept)	0.07	0.01–0.23	<0.001
Water plant coverage (%)	0.99	0.97–1.01	0.500
Woody debris (%)	1.06	1.03–1.10	<0.001
Canopy (%)	1.03	1.01–1.05	0.003

**Table 4:** Relative importance of each variable retained from the variable selection procedure. I: Percentage likelihood determined through hierarchical partitioning of each habitat variable contributing to the variation in total trapping success at each location.

Variable	Importance Rank	I (%)
Woody debris (%)	1	53.40
Canopy (%)	2	43.47
Water plant coverage (%)	3	3.13

**Table 5:** Comparison of the density of different softshell turtle species in previous studies and the current study.

Species and IUCN Red List Status	Density (/ha)	Area	Technique	References
*Pelodiscus maackii* (DD)	0.663/ha	Middle Ussuri River China side	Continuous-Time Capture–Recapture method	Present work
*Apalone mutica* (LC) and *Apalone spinifera* (LC)	0.11/ha for *Apalone mutica* and 0.33/ha for *Apalone spinifera*	Southern Illinois USA	Schumacher Eschmeyer method	[44]
*Apalone spinifera* (LC)	1.9/ha	Southeast Missouri USA	Closed population capture–recapture models using maximum-likelihood estimation	[45]
*Trionyx triunguis* (CR)	14/ha	Dalaman, southwestern Turkey	Jolly–Seber mark–recapture method	[38]
*Apalone ferox* (LC)	34.5/ha for adults and 18.4/ha for juveniles	Wekiwa Springs State Park, Florida, USA	Cormack–Jolly–Seber (CJS) models	[37]
*Apalone mutica* (LC)	42/ha	Kansas USA	The Jolly model	[46]

(DD), Data Deficient; (LC), Least Concern; (CR), Critically Endangered.

## Data Availability

Data will be made available upon request.
